# Development of an autophagy activator from Class III PI3K complexes, Tat-BECN1 peptide: Mechanisms and applications

**DOI:** 10.3389/fcell.2022.851166

**Published:** 2022-09-12

**Authors:** Yanfei He, Huaqing Lu, Yuting Zhao

**Affiliations:** Institute of Future Agriculture, Northwest A&F University, Yangling, China

**Keywords:** autophagy, drug development, Beclin 1, Class III PI3K complexes, Tat-BECN1 peptide, cell-penetrating peptides

## Abstract

Impairment or dysregulation of autophagy has been implicated in many human pathologies ranging from neurodegenerative diseases, infectious diseases, cardiovascular diseases, metabolic diseases, to malignancies. Efforts have been made to explore the therapeutic potential of pharmacological autophagy activators, as beneficial health effects from caloric restriction or physical exercise are linked to autophagy activation. However, the lack of specificity remains the major challenge to the development and clinical use of autophagy activators. One candidate of specific autophagy activators is Tat-BECN1 peptide, derived from Beclin 1 subunit of Class III PI3K complexes. Here, we summarize the molecular mechanisms by which Tat-BECN1 peptide activates autophagy, the strategies for optimization and development, and the applications of Tat-BECN1 peptide in cellular and organismal models of physiology and pathology.

## Introduction

Autophagy, the evolutionarily conserved pathway to target unwanted or damaged cellular contents to lysosomes for degradation, is linked to numerous human diseases (Levine and Kroemer, 2019; Mizushima and Levine, 2020) and considered a therapeutic target (Galluzzi et al., 2017).

Autophagy can be activated by many approaches, ranging from amino acid starvation, caloric restriction, physical exercises, to treatment with chemicals by known or unknown mechanisms (Galluzzi et al., 2017). Some chemicals that induce autophagy are under clinical trials for neurodegenerative diseases, cancers, autoimmune disorders or metabolic diseases, including (but not limited to) rapamycin, idalopirdine, SB-742457, metformin, resveratrol, lithium, spermidine, and trehalose (Galluzzi et al., 2017; Mizushima and Levine, 2020; Suresh et al., 2020). Among these autophagy activators, rapamycin is widely used in basic and translational research; it inactivates mechanistic target of rapamycin complex 1 (mTORC1), the nutrient-sensing kinase complex that promotes anabolism and inhibits catabolism, mainly autophagy (Saxton and Sabatini, 2017). However, specific autophagy activators that have no or limited impact on other pathways are lacking for research needs and for potential clinal use.

In 2013, Beth Levine lab reported an autophagy-inducing Tat-BECN1 peptide (hereinafter referred as to Tat-BECN1), derived from Beclin 1 region that is required for HIV-1 Nef binding (Shoji-Kawata et al., 2013). Beclin 1 is a subunit of both Class III PI3K complexes, where Class III PI3K-C1 is regarded as autophagy initiation complex for autophagosome nucleation, and Class III PI3K-C2 mediates autophagosome maturation, endocytosis and LC3-associated phagocytosis (LAP) (Levine et al., 2015; Hurley and Young, 2017). Upon Tat-BECN1 treatment, autophagy induction was observed in 10 cell lines and primary cultures (Table 1); when Tat-BECN1 was administrated to mice intraperitoneally, autophagy induction was observed in heart, skeletal muscle (vastus lateralis) and pancreas (Shoji-Kawata et al., 2013). Tat-BECN1 showed protective effects in several *in vitro* and *in vivo* infection models of Sindbis virus, chikungunya virus, West Nile virus (WNV), HIV-1, *L. monocytogenes* (Shoji-Kawata et al., 2013). It also enhanced the degradation of small mutant huntintin protein aggregates in cells, indicating a therapeutic potential for neurodegenerative diseases (Shoji-Kawata et al., 2013).

**TABLE 1:** Tat-BECN1 peptide-responsive cells and tissues

Species	Origins	Cells	References
Rat	Embryonic cardiac myoblast	H9c2	(Misaka et al., 2018)
Human	Cardiomyocyte	AC16	(Sun et al., 2021)
Rat, mouse, human iPSC-derived	Cardiomyocytes	Primary culture	(Nah et al., 2020)
Rat	Cortical neurons	Primary culture	(He et al., 2016)
Rat	Cerebral endothelial cells	Primary culture	(Forte et al., 2020)
Mouse	Cranial neural crest cells	Primary culture	(Yang et al., 2021)
Rat	Retinal neuron	R28	(Mathew et al., 2021)
Human	trabecular meshwork cells	Primary culture	(Kasetti et al., 2021)
Human	Kidney proximal tubule epithelial cell	HK-2	(Wang S. et al., 2015; Iaconis et al., 2020; Wang S. et al., 2021)
Opossum	Kidney proximal tubule epithelial cell	OKP	(Shi et al., 2020)
Mouse	Kidney proximal tubular cells	Primary culture	(Livingston et al., 2016)
Rat	Chondrosarcoma	RCS	(Bartolomeo et al., 2017)
Human	Chondrocytes	Primary culture	(Wu et al., 2020)
Mouse	Bone marrow stromal cells	Primary culture	(Choi et al., 2018)
Mouse	Pancreatic α cell	αTC9	(Rajak et al., 2021)
Rat	Insulinoma	INS-1E	(Riahi et al., 2016;Israeli et al., 2018)
Human	Breast cancer (Her2-positive)	BT-474, SK-BR3, MDA-MB-361	(Vega-Rubín-de-Celis et al., 2018)
Mouse	Breast cancer	4T1	(Wang et al., 2015b)
Human	Breast cancer (triple negative)	MDA-MB-231	(Zhou et al., 2019)
Mouse	Neuroblastoma	Neuro-2A	(Luo et al., 2018)
Human	Neuroblastoma	SK-N-SH	(Kobayashi et al., 2014;Kobayashi et al., 2020)
Human	Hepatocellular carcinoma	HepG2	(Wang et al., 2015b)
Human	Melanoma	WM793	(Kraya et al., 2015)
Human	Teratocarcinoma	NTERA-2/D1	(Sharif et al., 2017)
Human	Ovarian cancer	SKOV3	(Ding et al., 2018)
Human	Colon cancer	HCT116	(Andrejeva et al., 2020)
Human	Myeloid leukemia	U937	(Sharma et al., 2021)
Human	CD4+ T cells	Primary culture	(Zhang et al., 2019)
Mouse	CD8+ T cells	Primary culture	(Ko et al., 2021)
Human	Endothelial progenitor cells	Primary culture	(Forte et al., 2020)
Mouse	Bone marrow-endothelial progenitor cells	Primary culture	(Jiang et al., 2020)
Human	Embryonic kidney	HEK293	(Frudd et al., 2018)
Human	Bone osteogenic sarcoma	U2OS	(Wang et al., 2018)
African green monkey	Kidney	Vero-B4	(Gassen et al., 2019)
Chinese hamster	Ovary	CHO	(Braasch et al., 2021)
Tissues in which Tat-BECN1-induced autophagy is experimentally validated			
Species	Tissues	References	
Mouse	Heart	(Shirakabe et al., 2016;An et al., 2017;Sun et al., 2018;Tong et al., 2019;Nah et al., 2020)	
Rat, mouse	Brain	(Li et al., 2016;He et al., 2017;Zhang et al., 2017;Luo et al., 2018;Shehata et al., 2018;Glatigny et al., 2019;He et al., 2019;De Risi et al., 2020;Forte et al., 2020;Kim et al., 2021)	
Mouse	Spinal cord	(He et al., 2016)	
Mouse	Eye	(Kasetti et al., 2021)	
Mouse	Kidney	(Livingston et al., 2019;Shi et al., 2020)	
Mouse	Liver	(Soria et al., 2018;Soria et al., 2021)	
Mouse	Lung	(Nikouee et al., 2021)	
Mouse	Bone	(Cinque et al., 2015;Bartolomeo et al., 2017)	
Rat	Articular cartilage	(Wang F. et al., 2019)	
Mouse	Cartilage and synovium	(Rockel et al., 2020)	
Mouse	Ovary	(Watanabe et al., 2020)	
Mouse	Tumor xenografts	(Wang et al., 2015a; Wang et al., 2015b;Pietrocola et al., 2016;Ding et al., 2018;Vega-Rubín-de-Celis et al., 2018;Zhou et al., 2019)	
Zebrafish	Embryo	(Zhu et al., 2017)	
Cells in which Tat-BECN1 induces certain effects (autophagy induction not experimentally validated)			
Species	Origins	Cells	References
Mouse	Sinus nodal cells	Primary culture	(Woo and Kim, 2021)
Rat	Brain microvascular endothelial cells	Primary culture	(Forte et al., 2021)
Rat	Renal proximal tubular epithelial cells	Primary culture	(Forte et al., 2021)
Mouse	Embryonic carcinoma	P19	(Sharif et al., 2019)
Mouse	Macrophage-like	RAW 264.7	(Hadadi-Bechor et al., 2019)
Human	Acute myeloid leukemia	OCI-AML3	(Wang L. et al., 2019)
Mouse	Pancreatic islets	Primary culture	(Goginashvili et al., 2015)
Human	Lung fibroblast	Normal human lung fibroblasts (NHLFs)	(Sosulski et al., 2015)
Tissues in which Tat-BECN1 induces certain effects (autophagy induction not experimentally validated)			
Species	Tissues	References	
Mouse	Bladder	(Miao et al., 2015)	
Mouse	Gastrocnemius and Flexor Digitorum Brevis muscles	(Baraldo et al., 2020)	
Mouse	Orthotopic pancreas cancer of mouse PDAC cell line KPC	(Song et al., 2018)	

Cells and tissues in which Tat-BECN1-induced autophagy is reported in (Shoji-Kawata et al., 2013).

Cell lines: Human cervical cancer cell line HeLa, breast cancer cell line MCF-7, leukemia monocytic cell line THP-1, lung adenocarcinoma cell line HCC827 and A549, bronchial epithelial cell line HBEC30-KT, African green monkey kidney fibroblast-like cell line COS-7.

Primary cultures: human monocyte-derived macrophages (MDMs), murine bone marrow-derived macrophages (BMDMs), murine embryonic fibroblasts (MEFs).

Tissues: heart, skeletal muscle (vastus lateralis) and pancreas.

Cells in which Tat-BECN1-induced autophagy is experimentally validated.

In this review, we analyze the applications of Tat-BECN1 in around 100 studies since its discovery, discuss the current understanding of how it functions, the strategies to improve its activity, and the impact of Tat-BECN1 on pathophysiology. The modifications of Tat-BECN1 are discussed in details in OPTIMIZATION AND DEVELOPMENT session; in other sessions, Tat-BECN1 is used to refer to the agent, regardless of being the original or modified version.

## TAT-BECN1 and mechanisms of action

**FIGURE 1:**
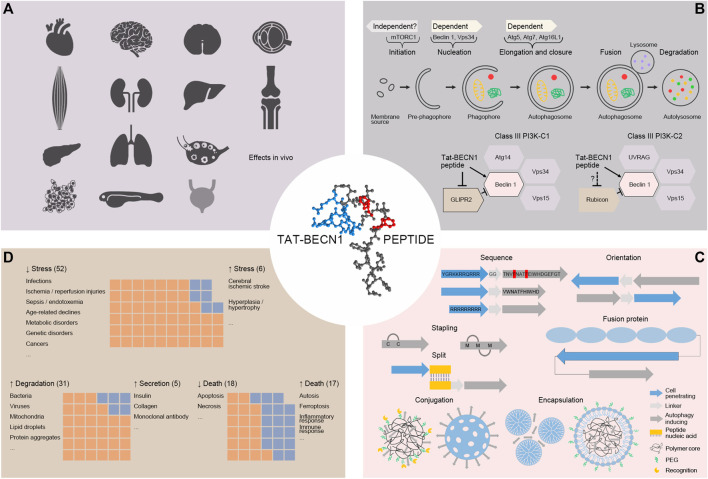
**(A)**. Autophagy induction by Tat-BECN1 peptide *in vivo*. In rodent heart, skeletal muscle, pancreas, brain, spinal cord, eye, kidney, liver, lung, bone, articular cartilage, ovary, cancer, as well as zebrafish embryo, Tat-BECN1 is reported to induce autophagy with experimental validation. For bladder (light grey), Tat-BECN1 induces effects but autophagy induction validation is not available. **(B)**. Molecular mechanisms of Tat-BECN1-induced autophagy. Tat-BECN1 functions in an autophagy gene-dependent and likely mTORC1-independent manner. Known protein targets include Beclin 1 in both Class III PI3K-C1 and -C2 complex and GLIPR2, a negative regulator of autophagy, binding to Beclin 1. **(C)**. Optimization of Tat-BECN1. Autophagy inducing element is from Beclin 1 BARA domain β-sheet 1 (18mer or 11mer) and hydrophobic residues in red are required for activity. To achieve cellular delivery, autophagy inducing element is fused or hybridized with cell penetrating element (in blue, Tat, oligoarginines, fusion protein), self-stapled, conjugated (polymer or silica based) or encapsulated (dendrimer based or lipid coated) with nanoparticles. **(D)**. The effects induced by Tat-BECN1 that impact pathophysiology. Graphs represent approximal numbers of studies showing indicated effects, i.e., alleviating stress (52) vs. augmenting stress (6), promoting degradation (31) vs. promoting secretion (5), inhibiting cell death (18) vs. promoting cell death (17).

Tat-BECN1 and modified versions have been employed by around 100 studies and shown robust induction of autophagy in a variety of cellular and organismal models. Tat-BECN1 can be directly applied to cultured cells, isolated tissues and embryos, injected intraperitoneally, intravenously, hippocampally, or infused directly to rodent animals. It is soluble in aqueous solution. Depending on cell or tissue type, the concentration and duration of treatment may vary. Other than the cells reported in the original study (Shoji-Kawata et al., 2013), around 40 cell lines or primary cell cultures respond to Tat-BECN1 for autophagy activation by experimental validation (Table 1). In addition to cardiac muscle, skeletal muscle and pancreas tissues reported in the original study (Shoji-Kawata et al., 2013), rodent heart (Shirakabe et al., 2016; An et al., 2017; Sun et al., 2018; Tong et al., 2019; Nah et al., 2020), brain (intraperitoneal injection (Li et al., 2016; He et al., 2017; Zhang et al., 2017; Forte et al., 2020; Kim et al., 2021), hippocampal injection (Glatigny et al., 2019; De Risi et al., 2020), intravenous injection (Luo et al., 2018), direct infusion (Shehata et al., 2018), intracerebroventricular injection (He et al., 2019)), spinal cord (He et al., 2016), eye (eye drop) (Kasetti et al., 2021), kidney (Livingston et al., 2019; Shi et al., 2020), liver (Soria et al., 2018; Soria et al., 2021), lung (Nikouee et al., 2021), bone (Cinque et al., 2015; Bartolomeo et al., 2017), articular cartilage (intra-articular injection) (Wang F. et al., 2019; Rockel et al., 2020), ovary (Watanabe et al., 2020), tumor xenografts of breast cancer cells (Wang et al., 2015a; Wang et al., 2015b; Vega-Rubín-De-Celis et al., 2018; Zhou et al., 2019), fibrosarcoma cells (Pietrocola et al., 2016) or ovarian cancer cells (Ding et al., 2018), as well as zebrafish embryos (Zhu et al., 2017), show increased autophagy level upon Tat-BECN1 treatment (Table 1; Figure 1A). Table 1 also summarizes cells and tissues that show responses to Tat-BECN1 while autophagy induction is not tested.

To understand the molecular mechanisms by which Tat-BECN1 exerts its effects will not only help elucidate the functionality of autophagy activation in physiology and pathology, but also shed light on how autophagy activators can be further optimized and designed. Here we discuss the progress of mechanistic studies of Tat-BECN1 (Figure 1B).

### Dependency of autophagy genes

Tat-BECN1-induced autophagy requires essential autophagy genes. Depletion of Beclin 1, Atg5 or Atg7, or inhibition of Class III PI3K complexes Vps34 lipid kinase activity by 3-methyladenine (3-MA) reduces Tat-BECN1-induced autophagy (Shoji-Kawata et al., 2013).

Essential autophagy genes are also required for Tat-BECN1-induced effects. Deletion of Atg5 abolishes the beneficial effects of Tat-BECN1, for instance, antiviral effect in human MDMs and the antibacterial effect in murine BMDMs (Shoji-Kawata et al., 2013), chronological lifespan extension in budding yeast cells (Plummer and Johnson, 2019), suppression of neutrophil infiltration into acutely inflamed mouse tissues (Reglero-Real et al., 2021). Tat-BECN1 mitigates myocardial reperfusion injury in wild type but not in cardiomyocyte-specific Atg7 knockout mice (Xie et al., 2021). Tat-BECN1 selectively eliminates memory CD4^+^ T cells with latent HIV infection, in an Atg5 and Atg7-dependent manner (Zhang et al., 2019). Tat-BECN1 differentially regulates lipid droplets degradation in macrophages depending on Atg7 and Atg12 (Hadadi-Bechor et al., 2019). Deletion of Atg16L1 decreases Tat-BECN1-induced degradation of mutant huntingtin aggregate in HeLa cells (Pavel et al., 2016). Inhibition of Class III PI3K complexes reverses Tat-BECN1-induced endothelial progenitor cells survival in oxygen and glucose deprivation conditions (Jiang et al., 2020).

### Dependency of mTORC1 pathway

Tat-BECN1 is widely regarded as an mTORC1-independent autophagy activator; however, experimental validation is limited. In HCT116 cells, Tat-BECN1 treatment does not affect the phosphorylation status of RPS6, a downstream target of mTORC1 (Andrejeva et al., 2020); in mouse pancreatic α cell line, Tat-BECN1 treatment does not affect the phosphorylation status of mTORC1 substrate RPS6KB1 (Rajak et al., 2021). Compared to inhibition of mTORC1 by rapamycin or Torin, Tat-BECN1 shows distinct effects on endothelial progenitor cells survival (Jiang et al., 2020), on glucagon degradation in pancreatic α cells (Rajak et al., 2021) and on blood glucose level in aged food-restricted mice (Nahata et al., 2021). Moreover, Tat-BECN1 prevents mitochondrial dysfunction and the appearance of markers of muscle fiber denervation caused by prolonged mTORC1 loss (rapamycin-treated Raptor-deleted muscles) (Baraldo et al., 2020), suggesting that Tat-BECN1 is likely to function independent of mTORC1. Extensive examination of the relationship between Tat-BECN1 and mTORC1 pathway is urgently needed.

### Protein targets

The cellular targets of Tat-BECN1 remain elusive. Efforts were made to identify proteins interacting with Tat-BECN1 by proteomic screen (Shoji-Kawata et al., 2013). On such list, GLIPR2, an evolutionarily conserved CAP protein superfamily member (Eberle et al., 2002), interacts with Tat-BECN1 and Beclin 1 via the peptide region (amino acids 267-284) (Shoji-Kawata et al., 2013; Li et al., 2017). GLIPR2 sequesters Beclin 1 in the Golgi apparatus; in the presence of Tat-BECN1, Beclin 1 is released from the Golgi pool for activation (Shoji-Kawata et al., 2013). However, GLIPR2 is not the sole target of Tat-BECN1 which can induce autophagy in GLIPR2 KO HeLa cells (Zhao et al., 2021). Other proteins that interact with both Tat-BECN1 and Beclin 1 include endosomal toll-like receptors TLR9 and TLR7 (Liu et al., 2020). Whether they play a role in Tat-BECN1-induced autophagy is yet to be examined.

Structural analyses of Class III PI3K complexes revealed that the first β-sheet of the Beclin 1 BARA (β-α repeated, autophagy-specific) domain is critical for membrane targeting of the complexes because this region (amino acids 265-287), which Tat-BECN1 falls into, is more accessible when the complexes are associated with liposomes in hydrogen deuterium exchange (HDX) assays (Rostislavleva et al., 2015; Ohashi et al., 2020). Two separate studies demonstrated that Tat-BECN1 increases Class III PI3K complexes kinase activity as well as membrane association on giant liposomes by fluorescence microscopy (Chang et al., 2019b) or on small liposomes by mass spectrometry and biochemical analyses (Zhao et al., 2021). The effects of Tat-BECN1 on Class III PI3K complexes require the hydrophobic residues (F270 and F274) (Chang et al., 2019b; Zhao et al., 2021), which are also required for autophagy induction (Shoji-Kawata et al., 2013; Peraro et al., 2017). Thus, Beclin 1 in Class III PI3K complexes is a direct target of Tat-BECN1. A domain-swapping model speculates that Tat-BECN1 competes with Beclin 1 BARA β-sheet 1 in binding with the rest of BARA domain and promotes β-sheet 1 for membrane association (Chang et al., 2019a).

Of note, as Tat-BECN1 targets Beclin 1, it may affect autophagy-independent Beclin 1 and/or Class III PI3K complexes functions, such as endocytosis. At a concentration which is not sufficient to induce autophagy, Tat-BECN1 promotes the endocytosis and degradation of tight junction protein occludin in an Atg16L1-indepentdent manner, and increases tight junction barrier permeability in human intestinal epithelial cells or mouse colons (Wong et al., 2019). At low dose, Tat-BECN1 increases the transduction of HIV-1-derived lentiviral vectors to human colorectal carcinoma cell line HCT116, and human hematopoietic stem and progenitor cells hCD34+ by enhancing viral fusion, in a Class III PI3K complex activity-independent manner (Majdoul et al., 2017). Therefore, before using Tat-BECN1 as an autophagy activator to assess the effect of autophagy induction on certain biological process, the treatment conditions shall be carefully evaluated and the dependency of autophagy genes shall be tested. Tat-BECN1 can also be further optimized to trigger autophagy-independent activities of Beclin 1 or Class III PI3K complexes.

## Optimization and development

On-going optimization and development of Tat-BECN1 aims to improve activity, solubility, membrane permeability and stability, as well as targeted delivery (Figure 1C).

### Optimization on Tat-BECN1

The original version of Tat-BECN1 consists of an HIV-1 Tat protein transduction domain amino acids 47–57 (YGRKKRRQRRR), a diglycine linker (GG) and a modified human Beclin 1 BARA β-sheet 1 (TNVFNATFEIWHDGEFGT); the H275E, S279D and Q281E substitutions from Beclin 1 amino acids 267–284 (TNVFNATFHIWHSGQFGT) are designed to enhance solubility (Shoji-Kawata et al., 2013). A later version of Tat-BECN1 is 7 amino acids shorter (also referred to as Tat-11mer, YGRKKRRQRRR-GG-VWNATFHIWHD) and two-fold more potent than the original version in cultured cells (Peraro et al., 2017). An alanine scan revealed that three residues corresponding to human Beclin 1 F270, F274 and I276 are essential for Tat-BECN1 activity, which are also evolutionarily conserved from yeast, fruit fly to mammals (Shoji-Kawata et al., 2013; Peraro et al., 2017).

Analogs of Tat-BECN1 are active with improved features. The retro-inverso Tat-BECN1 D-amino acid sequence is RRRQRRKKRGY-GG-TGFEGDHWIEFTANFVNT, with higher activity and resistance to proteolytic degradation *in vivo* compared to l-amino acids peptide (Shoji-Kawata et al., 2013). The retro-inverso version of Tat-11mer RRRQRRKKRGY-GG-DHWIHFTANWV, is active *in vivo*, however, less potent than its l-amino acid counterpart (Peraro et al., 2017). Moving Tat to C-terminal (11mer-Tat, VWNATFHIWHD-GG-YGRKKRRQRRR) or replacing Tat with nona-Arginine (Arg9-11mer), greatly increases cell penetration and autophagy-inducing activity (Peraro et al., 2018).

### Alternatives to cell-penetrating peptides

Tat and oligoarginines cell-penetrating peptides are highly positive-charged and can interact with negative-charged cellular molecules non-specifically. To minimize the off-target and toxic effects associated with cell-penetrating peptides, different approaches are employed to make Tat-BECN1 Tat-free.

The first strategy involves diversity-oriented stapling, where two cysteines are introduced to the Beclin 1 peptide sequence and cross-linked by thiol bis-alkylation to produce a stapled active peptide DD5-o (Peraro et al., 2017), or three methionine are introduced to the sequence and cross-linked by methionine bis-alkylation to produce a bicyclic active peptide 7f (Qin et al., 2019). Interestingly, although Tat-BECN1 shows as a random coil in circular dichroism, the structure of DD5-o and the activity of retro-inverso variants of Tat-BECN1 suggest helical conformations may be adopted upon action (Peraro et al., 2017). The second strategy is to split Tat-BECN1 based on peptide nucleic acids (PNAs), where the cell-penetrating peptide and Beclin 1 peptide are conjugated to two complementary PNAs, respectively; the two PNA-peptides form hybrid to enter the cells and partially dissociate to release Beclin 1 peptide (Hakata et al., 2020). The PNA1-Arg8/PNA2-Beclin 1 peptide hybrid is more active in autophagy induction compared to Arg8-Beclin 1, without causing cytotoxicity (Hakata et al., 2020). The third strategy takes advantage of the formation of nanoparticles with Beclin 1 peptide conjugated or encapsulated. For instance, Beclin 1 peptide can be fused to amphiphilic poly (β-amino ester) (Wang et al., 2015a), cationic chitosan (Luo et al., 2018), or photothermal agent polydopamine (Zhou et al., 2019) to assemble into nanoparticles. Other elements can be introduced simultaneously to facilitate targeted delivery, which will be discussed in the next session. Beclin 1 peptide can be directly packaged into metal (Mn^2+^)-terpyridine based coordinative dendrimer and released after cellular internalization with high efficiency compared to 11mer-Tat (Ren et al., 2022). The last strategy develops soluble Beclin 1 peptide-containing fusion proteins, where thioredoxin (Trx) confers solubility and thermal stability, pH low insertion peptide (pHLIP) triggers the fusion protein translocation across the plasma membrane in a tumor acidic environment (Ding et al., 2018).

### Targeted delivery of beclin 1 peptide

Tat-BECN1 shows punctate subcellular localization, which is not seen for F270 mutant (Shoji-Kawata et al., 2013). However, it is not fully characterized what organelles Tat-BECN1 is localized to. Engineering on Tat-BECN1 may facilitate specific organelle-targeting. Tat-BECN1 is grafted onto mesoporous silica nanoparticles (MSNs, around 72 nm in diameter) for perinuclear ER-targeted delivery; when such MSNs are loaded with brefeldin A, ER-phagy is induced (Wang et al., 2018). The pH-sensitive poly (β-amino ester) conjugated Beclin 1 peptides self-assemble into micelle-like nanoparticles (P-Bec1) and accumulate in lysosomes after cellular uptake; P-Bec1 causes lysosome damage and cell death in breast cancer cells (Wang et al., 2015a). Whether the observed cell death is due to autophagy induction or lysosome impairment is yet to be determined. Injected P-Bec1 effectively accumulates in MCF-7 tumor xenografts and inhibits tumor growth, without affecting normal tissues (Wang et al., 2015a).

Cell type-specific or preferential delivery of Beclin 1 peptide can be achieved. As tumor tissues are weakly acidic, a fusion protein Trx-pHLIP-Beclin 1 is designed to specifically accumulate in weakly acidic conditions (pH 6.5); Trx-pHLIP-Beclin 1 induces autophagic cell death in breast and ovarian cancer cells and suppresses growth of ovarian cancer xenografts, without causing systemic toxicity (Ding et al., 2018). As integrin αvβ3-overexpressing cancer cells recognize RGD sequence, PPBR nanoparticles, composed of polydopamine nanoparticle conjugated with Beclin 1 peptide, polyethylene glycol (PEG) and cyclic RGD, improves photothermal killing efficacy in breast cancer cells in an autophagy-dependent manner; PPBR nanoparticles suppresses the growth of near-infrared irradiated breast cancer xenografts more efficiently than nanoparticles without RGD (Zhou et al., 2019). To capture extracellular amyloid β-peptide (Aβ) for autophagic clearance, a self-destructive nanosweeper is designed with cationic chitosan core, Beclin 1 peptide and PEG conjugated KLVFF sequence that recognizes and co-assembles with Aβ; the nanosweeper reduces Aβ-induced cytotoxicity in mouse neuroblastoma cells, clears Aβ in the brain of Alzheimer’s disease mouse model and rescues memory deficits (Luo et al., 2018). Tat-BECN1 encapsulated and lipid-coated hybrid PLGA (poly lactic-co-glycolic acid) nanoparticles are found to preferentially induce autosis in memory CD4^+^ T cells with latent HIV infection but not in uninfected cells (Zhang et al., 2019); the molecular mechanisms underlying the specificity of such nanoparticles are yet to be explored.

## Impact of TAT-BECN1 on pathophysiology

As discussed above, Tat-BECN1 is widely tested as an autophagy activator for basic and translational research (Table 1), and shows great therapeutic potential in cancers, neurodegenerative diseases, infectious diseases, injury recoveries, aging and so on. Here we try to compare the effects of Tat-BECN1 that impact pathophysiology from three aspects, through mediating autophagic degradation or autophagy-dependent secretion, through promoting autophagic cell death or preventing apoptotic cell death, and through alleviating stress or augmenting stress (Figure 1D), in order to offer a comprehensive view of Tat-BECN1 application across different pathophysiological models.

## Effects of Tat-BECN1 on degradation

Tat-BECN1 can exert effects by inducing autophagic degradation, including general autophagy and selective autophagy (i.e., xenophagy, virophagy, mitophagy, lipophagy, ER-phagy and aggrephagy). Tat-BECN1 enhances bacterial clearance, during uropathogenic *E. coli* infection in mouse bladder (Miao et al., 2015), *M. tuberculosis* infection in mouse bone marrow-derived macrophages (BMDMs) (Franco et al., 2017), *K. pneumoniae* infection in mouse lungs (Nikouee et al., 2021) and opportunistic infection of *M. tuberculosis* or *M. avium* in HIV-1-infected human macrophages (Sharma et al., 2021). Tat-BECN1 protects against viral infections, by inhibiting HIV replication in human monocyte-derived macrophages (MDMs) (Zhang et al., 2018) and memory CD4^+^ T cells (Zhang et al., 2019), and by inhibiting Middle East respiratory syndrome coronavirus (MERS-CoV) replication in VeroB4 cells (Gassen et al., 2019). Tat-BECN1 activates mitophagy in rodent heart and protects against pressure-overload-induced heart failure (Shirakabe et al., 2016) and high fat diet-induced diabetic cardiomyopathy (Tong et al., 2019), in kidney and protects against renal ischemia/reperfusion injury (Livingston et al., 2016; Livingston et al., 2019), in brain and kidney and prevents high salt diet-induced hypertension-related stroke occurrence (Forte et al., 2020); it prevents mitochondrial dysfunction in skeletal muscles and the appearance of markers of muscle fiber denervation caused by prolonged mTORC1 loss (Baraldo et al., 2020). Tat-BECN1 induces lipophagy of acetylated low density lipoprotein-induced lipid droplets in macrophages; interestingly, it increases the biogenesis of oleic acid-induced lipid droplets (Hadadi-Bechor et al., 2019). Tat-BECN1 triggers degradation of amyloid fibrils in Alzheimer’s disease (Luo et al., 2018) and middle-aged (De Risi et al., 2020) brains and rescues memory deficits, removes ubiquitinated protein aggregates induced by WNV infection (Kobayashi et al., 2020), relieves abnormal cytosolic and nuclear glycogen storage in liver with distal urea cycle disorders (Soria et al., 2021), clears glaucoma-causing mutant myocilin and reduces elevated intraocular pressure (Kasetti et al., 2021), enhances FAM134B-mediated ER-phagy of misfolded procollagen in chondrocytes (Forrester et al., 2019). Other than protein quality control, Tat-BECN1 promotes degradation of synaptic proteins and erasure of reconsolidation-resistant fear memories (Shehata et al., 2018), stabilizes microtubules in neurons by targeting microtubule-destabilizing protein and accelerates axon regeneration after spinal cord injury (He et al., 2016), lowers cardiac lipoprotein lipase and triglyceride accumulation and restores cardiac function in obese mice (An et al., 2017), increases ureagenesis in liver by providing key urea-cycle intermediates and improves ammonia clearance in hyperammonemia model (Soria et al., 2018), reverts the accumulation of oxidized proteins like CaMKII and sinus node dysfunction induced by insulin sensitizers (Woo and Kim, 2021), downregulates β-catenin pathway and regulates chondrogenic fate specification of cranial neural crest cells (Yang et al., 2021) while downregulates Notch1 which counteracts β-catenin in bone marrow stromal cells (Choi et al., 2018), decreases the level of pluripotency factors POU5F1, NANOG and SOX2 in cancer stem cells (Sharif et al., 2017), decreases fibrotic markers and inhibits lung fibroblast to myofibroblast differentiation (Sosulski et al., 2015).

Emerging evidence indicates that autophagy pathway regulates secretion of specific cargos instead of lysosomal degradation (New and Thomas, 2019). Tat-BECN1 increases the release of insulin in murine and human primary islets (Goginashvili et al., 2015) (Tat-BECN1 is also reported to reduce adenosine-stimulated insulin secretion in islets (Israeli et al., 2018)), the secretion of candidate protein biomarkers of tumor cell autophagy (Kraya et al., 2015), and the production of monoclonal antibody in CHO cells (Braasch et al., 2021). Tat-BECN1 restores collagen secretion from chondrocytes to extracellular matrix and rescues bone growth in mice with lysosomal storage disorders (Bartolomeo et al., 2017) or mice with FGF signaling defects (Cinque et al., 2015). Tat-BECN1 attenuates extracellular matrix degradation in osteoarthritis chondrocytes (Wang F. et al., 2019; Wu et al., 2020) and ameliorates cartilage degeneration in rodent osteoarthritis model (Wang F. et al., 2019). Future work is needed to elucidate what mechanisms determine the destination of such cargos.

## Effects of Tat-BECN1 on cell death

Prolonged Tat-BECN1 treatment leads to autosis, an autophagy-dependent, apoptosis and necrosis-independent cell death, mediated by Na^+^/K^+^ ATPase pump (Liu et al., 2013; Fernández et al., 2020). Tat-BECN1 induces autosis in HIV-infected human MDMs (Zhang et al., 2018) and memory CD4^+^ T cells (Zhang et al., 2019), but not in uninfected counterparts; it sensitizes primary renal tubular cells to TGFβ1-induced non-apoptotic cell death (Livingston et al., 2016). Tat-BECN1 has been assessed for systemic toxicity *in vivo*, which is negligible (Shoji-Kawata et al., 2013; Wang et al., 2015a; Ding et al., 2018). However, it exhibits cytotoxicity in cancer cells (Wang et al., 2015a; Ding et al., 2018; Song et al., 2018; Zhou et al., 2019) and cancer stem-like cells (Sharif et al., 2017; Sharif et al., 2019), reduces the growth of tumor xenografts *in vivo* (Wang et al., 2015a; Ding et al., 2018; Song et al., 2018; Vega-Rubín-De-Celis et al., 2018; Zhou et al., 2019). Tat-BECN1 worsens the brain damage after cerebral ischemic stroke (He et al., 2017; Zhang et al., 2017; He et al., 2019) and abolishes the neuroprotective effects of such agents as ganglioside GM1 (Li et al., 2016), puerarin (He et al., 2017) and breviscapine (Zhang et al., 2017), likely due to aggravating inflammatory responses (He et al., 2019). What confer the sensitivity of HIV-infected cells, cancer cells and ischemic neurons to Tat-BECN1-induced cytotoxicity? How does Tat-BECN1 affect tumor growth, by autosis, ferroptosis (Song et al., 2018) or T cell-mediated immune response (Pietrocola et al., 2016)? Can Tat-BECN1 be modified for targeted delivery? Future research will be required to address these questions.

When autosis is already induced in cardiomyocytes during ischemia/reperfusion, Tat-BECN1 exacerbates myocardial injury (Nah et al., 2020); while administration of Tat-BECN1 prior to ischemia/reperfusion (Nah et al., 2020) or at the time of reperfusion (Xie et al., 2021) reduces cell death and protects cardiac function. This suggests that the timing of intervention is important; autophagy activation at early stage of a developing condition may be beneficial. Tat-BECN1 inhibits apoptotic cell death in many *in vitro* or *in vivo* models: during renal ischemia/reperfusion, pretreatment with Tat-BECN1 inhibits apoptosis and prevents renal injury (Livingston et al., 2019); under oxygen glucose deprivation, the *in vitro* model of ischemia, Tat-BECN1 decreases apoptosis of endothelial progenitor cells (Jiang et al., 2020) and retinal neurons (Mathew et al., 2021); Tat-BECN1 blocks cardiomyocytes apoptosis under pressure overload (Shirakabe et al., 2016); it also alleviates cisplatin (a cancer drug known for nephrotoxicity)-induced cell death in renal proximal tubule cells (Wang S. et al., 2021) or PKM2 silence-induced apoptosis in acute myeloid leukemia cell line with mutated nucleophosmin (Wang L. et al., 2019). Tat-BECN1 not only reduces apoptosis but enhances proliferation in synovial intima cells leading to synovial hyperplasia in mouse knee joints (Rockel et al., 2020); it augments cardiac hypertrophy in autosomal dominant polycystic kidney disease mouse model (Atwood et al., 2020). Tat-BECN1 attenuates apoptosis and necrosis, in brain endothelial cells and renal epithelial cells exposed to high salt (Forte et al., 2021), in human neuroblastoma cells infected with WNV (Kobayashi et al., 2020). Tat-BECN1 preserves brain structures and sensorimotor functions after neonatal hypoxic-ischemic injury (Kim et al., 2021), protects renal proximal tubule cells against phosphotoxicity induced by high phosphate and increases urinary phosphate excretion in mice (Shi et al., 2020), rescues Aldehyde dehydrogenase 5a1-deficient mice from premature lethality (Vogel et al., 2016), increases survival rate of zebrafish embryos with anthracycline-induced cardiotoxicity (Wang Y. et al., 2021). Administration of Tat-BECN1 at neonatal stage upregulates the number of primordial follicles even at middle-age and extends the fertility and reproductive lifespan of female mice (Watanabe et al., 2020).

## Effects of Tat-BECN1 on stress

Autophagy is a major pathway to respond to stress and maintain homeostasis. In most examples discussed in the sessions above, Tat-BECN1 exhibits beneficial effects to cells or organisms under stress conditions, like protections against bacterial and viral infections, recoveries from injuries, detoxication of aberrant metabolism. A few exceptions include that Tat-BECN1 increases unexpected synovial hyperplasia (Rockel et al., 2020) and cardiac hypertrophy (Atwood et al., 2020); it intensifies brain damage after cerebral ischemic stroke (Li et al., 2016; He et al., 2017; Zhang et al., 2017; He et al., 2019). On the contrary, in neonatal hypoxia-ischemia model (Kim et al., 2021) or high salt diet-induced hypertension-related stroke model (Forte et al., 2020), Tat-BECN1 prevents brain damage; it also improves the formation of long-term spatial memory (Hylin et al., 2018) or novel memory (Glatigny et al., 2019) in young rodents, rescues age-related memory decline in middle-aged mice (Glatigny et al., 2019; De Risi et al., 2020). How brain tissues respond to Tat-BECN1 treatment in different models shall be further investigated.

In addition to the abovementioned scenarios, during endotoxemia induced by lipopolysaccharide (LPS) or sepsis induced by pneumonia, Tat-BECN1 alleviates inflammation and reduces injuries in heart (Sun et al., 2018; Sun et al., 2021) and lung (Nikouee et al., 2021); it suppresses neutrophil infiltration into acutely inflamed mouse tissues (Reglero-Real et al., 2021). In undernourished aged mice, Tat-BECN1 decreases the plasma levels of the glucogenic amino acid and restores the blood glucose levels to maintain energy homeostasis (Nahata et al., 2021), in line with the beneficial effects of Tat-BECN1 in age-related decline in memory (Glatigny et al., 2019; De Risi et al., 2020) or fertility (Watanabe et al., 2020). Tat-BECN1 mediates cell differentiation and fate determination (Sosulski et al., 2015; Sharif et al., 2017; Choi et al., 2018; Yang et al., 2021). It is reported to affect the length of primary cilia; however, the effect is controversial (Wang S. et al., 2015; Iaconis et al., 2020; Wang S. et al., 2021).

## Discussion

As discussed above, Tat-BECN1 is a potent autophagy activator and a powerful tool to investigate the role of autophagy in various cells and tissues; optimal treatment conditions, especially timing, concentration/dosage and duration, shall be tested in cells or tissues of interest beforehand. Tat-BECN1 is likely to function independent of mTORC1 and a side-by-side comparison of Tat-BECN1 and mTORC1 inhibitors is recommended.

A thorough understanding of the molecular mechanisms underlying Tat-BECN1-induced autophagy is of importance. So far, GLIPR2 (Shoji-Kawata et al., 2013; Zhao et al., 2021) and Beclin 1 in Class III PI3K complexes (Chang et al., 2019b; Zhao et al., 2021) are experimentally confirmed Tat-BECN1 targets. GLIPR2 is a negative regulator of autophagy interacting with Tat-BECN1 and Beclin 1 via BARA domain β-sheet 1 (Shoji-Kawata et al., 2013; Li et al., 2017; Zhao et al., 2021); it will be interesting to test whether Rubicon, a well-known negative regulator of autophagy, which also interacts with Beclin 1 via BARA domain β-sheet 1 (Chang et al., 2019b), is another target of Tat-BECN1. Class III PI3K-C1 (autophagy initiation) and -C2 (autophagosome maturation, endocytosis, LAP) complexes (Levine et al., 2015; Hurley and Young, 2017) are not discerned by Tat-BECN1 for activation, at least *in vitro* (Chang et al., 2019b), which may explain why Tat-BECN1 triggers autophagy-independent effect such as endocytosis (Wong et al., 2019). Further investigation on Class III PI3K complexes and regulators may facilitate the design of Class III PI3K-C1 or -C2-specific Tat-BECN1.

Advancement in chemical biology and materials science will accelerate the optimization and development of Tat-BECN1 with better bioactivity and efficacy. Additional work is required to determine long-term and systemic effects of Tat-BECN1 treatment *in vivo*, before moving forward to test Tat-BECN1 as novel clinical therapeutics.
